# Herpesviridae lung reactivation and infection in patients with severe COVID-19 or influenza virus pneumonia: a comparative study

**DOI:** 10.1186/s13613-022-01062-0

**Published:** 2022-09-24

**Authors:** Charles-Edouard Luyt, Sonia Burrel, David Mokrani, Marc Pineton de Chambrun, Domitille Luyt, Juliette Chommeloux, Vincent Guiraud, Nicolas Bréchot, Matthieu Schmidt, Guillaume Hekimian, Alain Combes, David Boutolleau

**Affiliations:** 1 Service de Médecine Intensive Réanimation, Institut de Cardiologie, Assistance Publique–Hôpitaux de Paris (APHP), Sorbonne-Université, Groupe Hospitalier Pitié, Salpêtrière, 47–83, Boulevard de l’Hôpital, 75651 Paris Cedex 13, France; 2 Sorbonne Université, INSERM, UMRS_1166-ICAN Institute of Cardiometabolism and Nutrition, Paris, France; 3 Centre National de Référence Herpèsvirus (Laboratoire Associé), Service de Virologie, AP-HP, Sorbonne Université, Hôpital Pitié-Salpêtrière, Paris, France; 4 Sorbonne Université, INSERM U1136, Institut Pierre Louis d’Epidémiologie et de Santé Publique (iPLESP), Paris, France

**Keywords:** Herpes simplex virus, Cytomegalovirus, COVID-19, Influenza, Acute respiratory distress syndrome

## Abstract

**Background:**

Lung reactivations of *Herpesviridae*, herpes simplex virus (HSV) and cytomegalovirus (CMV) have been reported in COVID-19 patients. Whether or not those viral reactivations are more frequent than in other patients is not known.

**Methods:**

Retrospective monocentric cohort study of 145 patients with severe COVID-19 pneumonia requiring invasive mechanical ventilation and who were tested for HSV and CMV in bronchoalveolar lavage performed during fiberoptic bronchoscopy for ventilator-associated pneumonia suspicion. Rates of HSV and CMV lung reactivations, and HSV bronchopneumonitis were assessed and compared with an historical cohort of 89 patients with severe influenza pneumonia requiring invasive mechanical ventilation.

**Results:**

Among the 145 COVID-19 patients included, 50% and 42% had HSV and CMV lung reactivations, respectively, whereas among the 89 influenza patients, 63% and 28% had HSV and CMV lung reactivations, respectively. Cumulative incidence of HSV lung reactivation (taking into account extubation and death as competing events) was higher in influenza than in COVID-19 patients (*p* = 0.03), whereas the rate of HSV bronchopneumonitis was similar in both groups (31% and 25%, respectively). Cumulative incidence of CMV lung reactivation (taking into account extubation and death as competing events) was similar in COVID-19 and influenza patients (*p* = 0.07). Outcomes of patients with HSV or CMV lung reactivations were similar to that of patients without, whatever the underlying conditions, i.e., in COVID-19 patients, in influenza patients, or when all patients were grouped.

**Conclusions:**

HSV and CMV lung reactivations are frequent in COVID-19 patients, but not more frequent than in patients with influenza-associated severe pneumonia, despite a higher severity of illness at intensive care unit admission of the latter and a longer duration of mechanical ventilation of the former. Although no impact on outcome of HSV and CMV lung reactivations was detected, the effect of antiviral treatment against these *Herpesviridae* remains to be determined in these patients.

**Supplementary Information:**

The online version contains supplementary material available at 10.1186/s13613-022-01062-0.

## Background

Viral reaction of herpes simplex virus (HSV) is common in the respiratory tract of mechanically ventilated patients, with frequency ranging from 30% to 60%, depending on case mix [1, 2]. Cytomegalovirus (CMV) blood reactivation is also frequent, occurring in 30% of seropositive intensive care unit (ICU) patients, whereas CMV lung reactivation/infection is less frequent [2–4]. Although several data suggest that *Herpesviridae* [HSV, CMV, but also Epstein–Barr virus (EBV) or human herpesvirus (HHV-6)] reactivation/detection in the blood and/or in the lower respiratory tract (LRT) is frequent in patients with severe coronavirus infectious disease-19 (COVID-19) pneumonia [5–10], it is not known whether these reactivations/infections/detections are more frequent in COVID-19 patients than in patients with pneumonia of other origins. Yet, it has been shown that patients with severe COVID-19 pneumonia hospitalized in the ICU and receiving mechanical ventilation (MV) are prone to develop bacterial or fungal ventilator-associated LRT infection with higher incidence rates than patients with pneumonia due to other causes [11–13]. Whether or not *Herpesviridae* reactivations are also more frequent in severe COVID-19 patients requiring MV, as compared to patients receiving MV for other causes, remains to be determined.

We, therefore, conducted a retrospective monocentric cohort study to evaluate frequencies and outcomes of HSV and CMV reactivations and infections in the LRT of patients with severe COVID-19 pneumonia, as compared to historical cohort of patients with severe influenza-associated pneumonia. Our hypothesis was that COVID-19 patients and influenza patients had similar rates of HSV and CMV reactivations.

### Methods

## Patients

All consecutive ICU-admitted patients, with confirmed COVID-19 pneumonia, based on reverse-transcriptase–polymerase-chain reaction (RT–PCR) performed on a respiratory specimen, between March 2020 and June 2021, and having received MV (MV), were included in this retrospective, monocenter, observational study. COVID-19 patients without MV were not included, because testing for *Herpesviridae* infection in the LRT of non-intubated patients is not routinely performed. Among screened patients, those having at least one lower respiratory tract sample tested for *Herpesviridae* (HSV and CMV) during their ICU stay were included. This group is hereafter called “COVID-19 group”.

Patients with confirmed influenza pneumonia admitted to our ICU between January 2013 and March 2020, having received invasive MV and having at least one LRT sample tested for *Herpesviridae* (HSV and CMV) served as controls (hereafter called “Influenza group”) [14].

### Procedures

In our ICU, mechanically ventilated patient clinically suspected of having developed ventilator-associated pneumonia (VAP) undergo fiberoptic bronchoscopy and bronchoalveolar lavage (BAL) [2, 15]. BAL fluid samples are processed in the bacteriological laboratory, looking for bacterial pneumonia, and in the virological laboratory, looking for viral reactivation/infection (HSV and CMV). The same procedures were performed in patients with COVID-19- and influenza-associated acute respiratory distress syndrome (ARDS).

Some patients had HSV or CMV blood testing at the time of BAL, the indication for HSV or CMV blood testing was driven by the physician in charge of the patient.

### Virological analysis

BAL fluid and whole blood samples were processed in the virology laboratory for HSV and CMV genome quantification. For BAL fluid samples, HSV and CMV genomes, together with albumin gene, were quantified using in-house real-time PCRs, as previously described, and viral loads were calculated and expressed in copies (for HSV) or international units (IU, for CMV) per million of cells collected by BAL [16, 17]. For whole blood samples, HSV and CMV genomes were quantified using HSV1&2 VZV R-GENE® kit (BIOMERIEUX) and artus® CMV QS-RGQ kit (Qiagen), respectively. Results were expressed in copies/mL (HSV) or in IU/mL (CMV).

### Outcomes

The primary outcomes were prevalence of HSV and CMV lung reactivations and HSV bronchopneumonitis in patients with COVID-19 and patients with influenza. Secondary outcomes were ICU length of stay and ICU mortality in both groups.

### Definitions

#### Herpesviridae reactivations

HSV and CMV LRT reactivations were defined as a positive PCR in BAL, for HSV or CMV, respectively, whatever the virus loads.

For patients with viral blood testing, HSV and CMV blood reactivations were defined as a PCR positive for the corresponding virus in blood, whatever the virus load.

#### HSV bronchopneumonitis

HSV bronchopneumonitis was defined as a clinical suspicion of VAP associated with a PCR positive for HSV with a virus load ≥ 10^5^ copies per million of cells. This cutoff was chosen according to previous publications, which found that it was associated with HSV bronchopneumonitis, diagnosed on histology or cytology [18]. Since no data exist regarding the relationship between CMV load in the LRT and CMV lung disease, this latter could not be defined in the present study.

#### ICU-acquired infections

VAP was diagnosed in patients having received MV for at least 48 h when the following two criteria were met: (1) clinically suspected VAP, defined as a new and persistent pulmonary infiltrate on chest radiograph associated with at least two of the following: temperature ≥ 38 °C, white blood cell count ≥ 10 Giga/L, purulent tracheal secretions, increased minute ventilation, arterial oxygenation decline requiring modifications of the ventilator settings and/or need for increased vasopressor infusion. For patients with ARDS, for whom demonstration of radiologic deterioration is difficult, at least two of the preceding criteria sufficed; and (2) significant quantitative growth (≥ 10^4^ colony-forming units/mL) of distal BAL fluid samples [19, 20].

Bloodstream infection (BSI) was defined as a bacterial infection identified on blood cultures. If coagulase-negative *Staphylococcus* were identified, it was considered as a BSI only if 2 sets of blood cultures grew with the same pathogen exhibiting the same resistance profile [21].

### Data collection and analysis

The following data were prospectively recorded in each patient’s medical chart: age, sex, presence of immunosupression (defined as one of the following condition: solid organ transplantation, hematological malignancy or treatment with immunosuppressant drug, including corticosteroids at a dose ≥ 0.5 mg/kg/d for ≥ 1 month). Simplified Acute Physiology Score (SAPS) II and Sequential Organ-Failure Assessment (SOFA) score at ICU admission, date COVID-19 or influenza symptoms started, date of hospital and ICU admission, date of MV onset, bacterial coinfection at ICU admission, presence or not of ARDS according to Berlin definition [22], need for extracorporeal membrane oxygenation (ECMO) device and its type (veno-venous or veno-arterial), episodes of bacterial VAP and episodes of BSI. ICU mortality was also recorded.

### Study endpoints

Study endpoints were rates of HSV lung reactivation, HSV bronchopneumonitis and CMV lung reactivation in COVID-19 and influenza patients. These frequencies were compared between COVID-19 and influenza patients. Secondary endpoint was the impact of HSV and CMV reactivations on ICU mortality in all patients and in each group separately.

### Statistical analyses

Data are expressed as median (interquartile range [IQR]) or n (%). Between-group comparisons were analyzed using Student’s *t* test or Mann–Whitney *U* tests according to variable’s distribution, i.e., normal or not, respectively, for continuous variables. Between-group differences were assessed with chi-square test or Fisher’s exact test for nominal variables. Incidences of HSV and CMV reactivations, as well as HSV and bronchopneumonitis in the 2 groups (primary outcomes) were compared using an estimated cumulative incidence function to take into account competing factors (death or extubation), as previously described [23]: cumulative incidence of viral reactivation, extubation and death were estimated in each group, taking into account only the first event, and compared. No sample size was calculated. Univariable analyses of factors associated with ICU mortality were performed. For this analysis, *Herpesviridae* reactivation was converted into a 4 categories variable: no viral reactivation, HSV reactivation, CMV reactivation, and both HSV and CMV reactivations (corresponding to patients having both reactivations). Thereafter, multiple logistic-regression analyses using backward-stepwise variable elimination were run (with the variable-exit threshold set at *p* < 0.05). Factors achieving *p* ≤ 0.10 in our univariable analyses were entered into the multivariable model. *Herpesviridae* reactivation was forced into the multivariable models. All potential explanatory variables included in the multivariable analyses were subjected to collinearity analysis with a correlation matrix. Variables associated with one another were not included in the model: because the SAPS II was strongly associated with the SOFA score, the former was not included in the final model. All reported *P* values are two-sided, and *p* < 0.05 was considered statistically significant. Analyses were computed using SPSS Version 23 (IBM SPSS, Chicago, IL) and R software, version 3.5.1 (R Foundation).

### Ethics

In accordance with current French law, informed written consent for demographic, physiologic and hospital-outcome data analyses was not obtained, because this observational study did not modify existing diagnostic or therapeutic strategies. Nonetheless, patients and/or relatives were informed about the anonymous data collection and told that they could decline inclusion. The database is registered with the Commission Nationale l’Informatique et des Libertés (CNIL, registration no. 1950673).

## Results

**Fig. 1:**
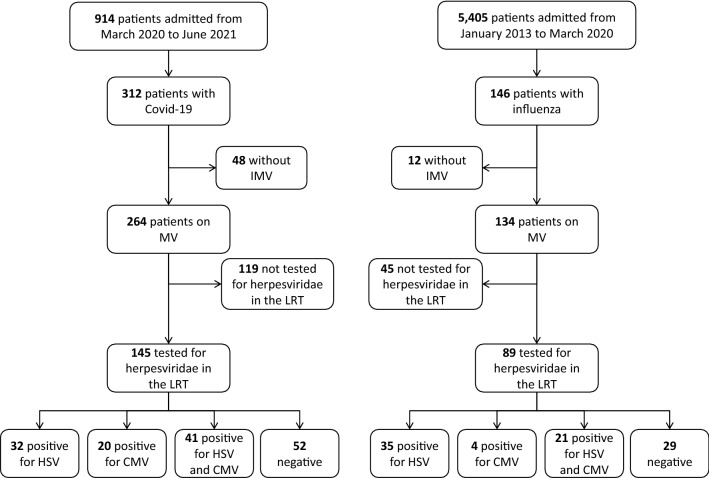
Flow chart of the study. *COVID-19*  coronavirus-infection disease 2019, *MV* mechanical ventilation, *HSV*  herpes simplex virus, *CMV* cytomegalovirus, *LRT* lower respiratory tract

During the study period, 312 patients were admitted to our ICU for COVID-19. Among them, 264 were mechanically ventilated, and 145 had at least one LRT sample tested for *Herpesviridae* (Fig. 1). Baseline characteristics and outcomes of these patients are given in Table 1. All were severely ill, requiring ECMO support for all except one; they had prolonged MV duration and ICU length of stay and their ICU-mortality rate was 43%.

**Table 1:** Characteristics of patients

Characteristic	COVID-19 patients (*n* = 145)	Influenza patients (*n* = 89)
*ICU admission*		
Age, y	53 (44–58)	55 (44–62)
Male sex^a^	103 (71)	50 (56)
Symptom-onset-to-ICU-admission interval, days^a^	7 (5–10)	8 (5–14)
Admission SAPS II^a,b^	59 (52–67)	71 (59–83)
Admission SOFA score^a,c^	12 (9–13)	15 (12–17)
Immunocompromised^d^	12 (8)	13 (15)
Documented bacterial coinfection^a^	10 (19)	41 (46)
*Procedures and outcome during ICU stay*		
Corticosteroids use^a,e^	127 (88)	18 (20)
Tocilizumab use^a^	13 (9)	0
ECMO^a^		
VA-ECMO	3 (2)	20 (22)
VV-ECMO	141 (97)	65 (73)
No ECMO	1 (1)	4 (5)
ICU-acquired infection		
Ventilator-associated pneumonia	134 (92)	53 (60)
Bloodstream infection	79 (54)	26 (29)
Days on ECMO^a^	30 (12–45)	13 (7–28)
Days on mechanical ventilation^a,b^	44 (24–62)	27 (13–48)
ICU length of stay, days^a^	49 (31–69)	26 (11–47)
ICU mortality rate, days	63 (43)	44 (49)

Results are expressed as median (IQR) or n (%). *COVID-19*  coronavirus-infection disease 19, *ICU* intensive care unit, *SAPS II*  severe acute physiology score, *SOFA*  sequential organ failure assessment, *VAP*  ventilator-associated pneumonia, *ARDS*  acute respiratory distress syndrome, *ECMO*  extracorporeal membrane oxygenation

^a^*p* < 0.05

^b^Possible score, 0 to 163; higher scores indicate greater disease severity; *p* < 0.0001

^c^Calculated from 6 variables obtained the day of admission, taking into account each parameter’s worst values during the 24 h following admission. Scores range from 0 to 24, with higher scores indicating more severe organ failure and higher mortality risk. Patients with a SOFA score = 10 have a 40–50% predicted mean chance of survival; *p* < 0.01

^d^Solid organ transplant recipients, hematological malignancy or receiving immunosuppressant drug (including corticosteroids at a dose ≥ 0.5 mg/kg/d for ≥ 1 month)

^e^At a dose ≥ 40 mg/d of prednisone or its equivalent for at least 5 consecutive days, and including high doses of methylprednisolone for persistent acute respiratory distress syndrome

Among the 146 patients with influenza admitted in our ICU during the 2013–2020 periods, 134 were mechanically ventilated, and 89 had at least one LRT sample tested for *Herpesviridae* (Fig. 1). Baseline characteristics and outcomes of these patients are displayed in Table 1. Among these 89 patients, 79 (89%) were infected with influenza A virus (subtypes H1N12009 for 62, H3N2 for 3, and not known for 14) and 10 (11%) with influenza B virus. Whereas influenza patients were sicker at ICU admission than COVID-19 patients, their duration of MV and ICU length of stay were shorter (Table 1). ICU-mortality rates were similar among the 2 groups.

### HSV lung reactivation and bronchopneumonitis

**Fig. 2:**
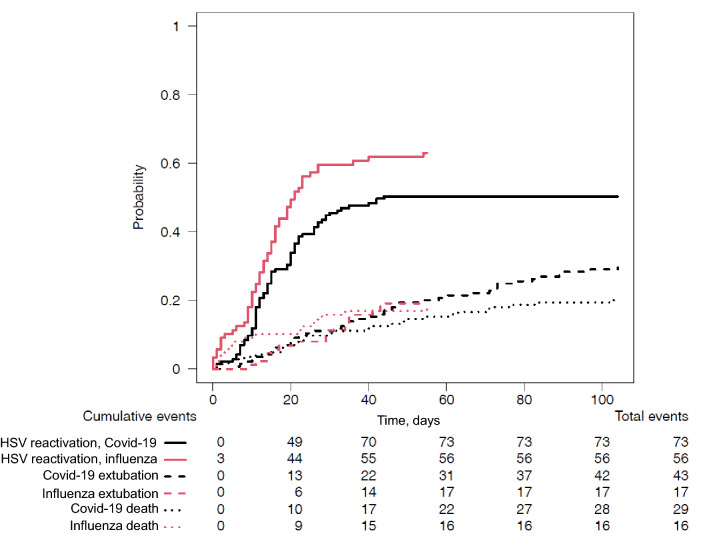
Estimated cumulative incidence of herpes simplex virus (HSV) lung reactivation, extubation or death in COVID-19 and influenza patients, taking into account only the first event that occurred. HSV reactivation refers to patients whose first event was HSV reactivation in lung; extubation refers to patients whose first event was extubation, and death refers to patients whose first event was death. *p* values for differences between COVID-19 and influenza patients were 0.03 for HSV reactivation, 0.53 for death and 0.87 for extubation

Although the proportion of patients with HSV lung reactivation was similar in patients with COVID-19 and influenza (50% and 63%, respectively, *p* = NS), estimated cumulative incidence of HSV reactivation (taking into account death and extubation as competing factors) was significantly higher in influenza patients than in COVID-19 patients (*p* = 0.03), whereas death and extubation did not differ between these 2 groups (*p* = 0.53 and 0.87, respectively) (Fig. 2). HSV reactivation occurred later in the COVID-19 group, but the highest virus loads among patients with HSV reactivation were similar in both groups (Table 2). Among the 73 COVID-19 patients with HSV lung reactivation, 32 (44%) were isolated and 41 (56%) were associated with CMV lung reactivation. Among the 53 influenza patients with HSV lung reactivation, 32 (60%) were isolated and 21 (40%) were associated with CMV lung reactivation (Table 3).

**Table 2:** Virological findings

Characteristic	COVID-19 patients *n* = 145	Influenza patients *n* = 89
HSV lung reactivation	73 (50)	56 (63)
Time from MV start to first HSV detection, days^a,b^	13 (10–21)	10 (6–15)
Peak of HSV load in BAL, copies/million cells^c^	58,312 (3363–3,144,816)	120,359 (6188–1,629,398)
HSV bronchopneumonitis^d^	36 (25)	28 (31)
Time from MV start to HSV bronchopneumonitis, days	15 (10–21)	14 (12–20)
CMV lung reactivation^a^	61 (42)	25 (28)
Time from MV to first CMV detection, days^a,b^	32 (26–42)	25 (16–39)
Peak of CMV load in BAL, IU/million cells^c^	1849 (241–6,460)	770 (293–3383)
Time from MV start to peak of CMV load in lung, days^a^	36 (28–49)	28 (16–42)

Results are expressed as n (%) or median (IQR). *COVID-19* coronavirus infectious disease 19, *HSV* herpes simplex virus, *BAL* bronchoalveolar lavage, *CMV* cytomegalovirus, *MV* mechanical ventilation, *IU* international unit

^a^*p* < 0.05 for between groups comparison

^b^Corresponding to the time between onset of mechanical ventilation and first detection of the virus (HSV or CMV) in bronchoalveolar lavage sample

^c^Corresponding to the highest virus load in patients with more than one sample

^d^Defined as a HSV virus load ≥ 10^5^ copies/million cells in BAL fluid

**Table 3:** Outcomes in COVID-19 and influenza patients according to *Herpesviridae* reactivations

	No *Herpesviridae* reactivation *N* = 81		HSV reactivation *N* = 67		CMV reactivation *N* = 24		HSV and CMV reactivation *N* = 62	
	COVID-19 *N* = 52	Influenza *N* = 29	COVID-19 *N* = 32	Influenza *N* = 35	COVID-19 *N* = 20	Influenza *N* = 4	COVID-19 *N* = 41	Influenza *N* = 21
Duration of MV, days	23 (13–47)	22 (9–34)	44 (32–56)	22 (14–53)	58 (35–75)	40 (23–53)	57 (44–67)	50 (20–69)
ICU length of stay, days	30 (19–50)	24 (9–35)	54 (39–70)	23 (10–55)	67 (47–89)	26 (15–42)	58 (47–72)	40 (17–73)
ICU mortality	21 (40)	13 (45)	12 (38)	17 (49)	8 (40)	3 (75)	22 (54)	11 (52)

Results are expressed as n (%) or median (IQR), as appropriate

*COVID-19* coronarovirus infectious disease 19, *HSV* herpes simplex virus, *CMV* cytomegalovirus, *MV* mechanical ventilation, *ICU* intensive care unit

**Fig. 3:**
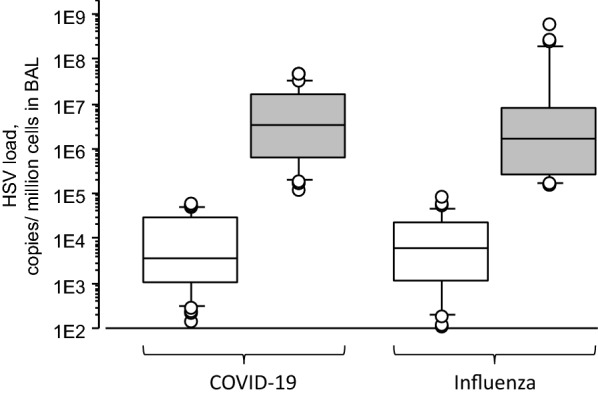
Herpes simplex virus (HSV loads in COVID-19 and influenza patients with (grey boxes) and without (white boxes) HSV bronchopneumonitis, respectively. The box plots report: the internal horizontal line is the median; the lower and upper box limits are the quartile 1 and quartile 3, respectively; bars represent the 95% confidence interval. The black circles are outliers

Proportion of patients developing HSV bronchopneumonitis was similar in COVID-19 and influenza patients, within similar time interval from MV start to HSV bronchopneumonitis diagnosis (Table 2) and with similar viral loads (Fig. 3).

Proportion of patients having received aciclovir (all because of virus load ≥ 10^5^ copies per million of cells, at a dosing of 10 mg/kg every 8 h during 10 to 15 days, dosing adjusted on renal function) was similar among COVID-19 and influenza patients; 35/145 (24%) and 27/89 (30%), respectively (*p* = 0.3).

### CMV lung reactivation

**Fig. 4:**
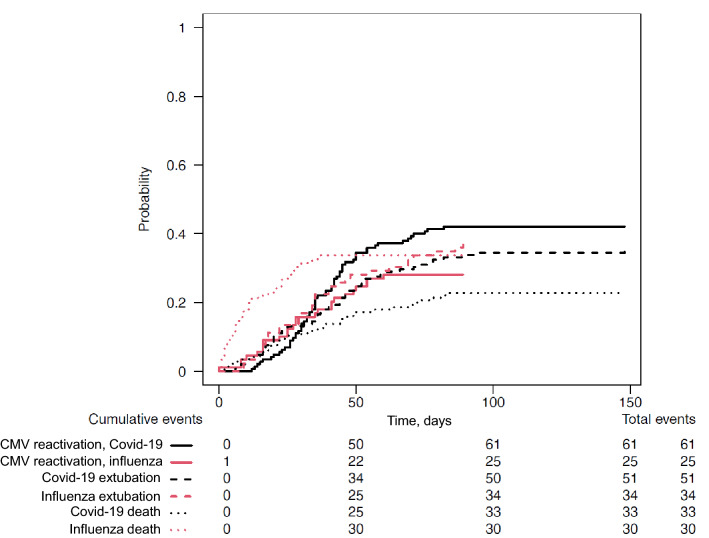
Estimated cumulative incidence of cytomegalovirus (CMV) lung reactivation, extubation or death in COVID-19 and influenza patients, taking into account only the first event that occurred. CMV reactivation refers to patients whose first event was CMV reactivation in lung; extubation refers to patients whose first event was extubation, and death refers to patients whose first event was death. *p* values for differences between COVID-19 and influenza patients were 0.07 for HSV reactivation, 0.03 for death and 0.49 for extubation

Although the proportion of patients with COVID-19 had more frequent CMV lung reactivation than patients with influenza (42% vs. 28%, *p* = 0.03), estimated cumulative incidence of CMV lung reactivation (taking into account death and extubation as competing factors) was similar in influenza patients and in COVID-19 patients (*p* = 0.07, see Fig. 4). CMV reactivation occurred later in COVID-19 patients but with a same peak in virus load (Table 2). Among the 61 COVID-19 patients with CMV lung reactivation, 41 (67%) had previous or concomitant HSV lung reactivation and 20 (33%) CMV reactivation alone. Among the 25 influenza patients with CMV lung reactivation, 21 (84%) had previous or concomitant HSV lung reactivation and 4 (16%) had CMV reactivation only (Table 3).

No patients received ganciclovir.

### HSV and CMV blood reactivation

Only few patients had HSV and CMV blood testing. Results are presented in the online supplement (Additional file 1: Table S1).

### Herpesviridae reactivation and immunosuppressive treatment

Among the COVID-19 patients, 13 received tocilizumab and none received an Il-1 inhibitor, whereas 127 (88%) received corticosteroids at a dosing ≥ 40 mg/day for at least 5 days (Table 1). The use of tocilizumab and corticosteroids were not associated with an increased risk of HSV or CMV reactivation: among the 93 COVID-19 patients who had at least one *Herpesviridae* reactivation (HSV, CMV or both), 11 (12%) received tocilizumab and 81 (88%) received corticosteroids (at a dosing ≥ 40 mg/day for at least 5 days), whereas among the 50 COVID-19 patients without any *Herpesviridae* reactivation, 2 (4%) received tocilizumab and 46 (88%) received corticosteroids (*p* = 0.18 for tocilizumab and *p* > 0.99 for corticosteroids).

Similar results were found regarding influenza patients: none received tocilizumab or another anti-interleukine-6 or anti-interleukine-1; but among the 60 patients who had at least one *Herpesviridae* reactivation (HSV, CMV or both), 10 (17%) received corticosteroids, whereas among the 29 patients without any *Herpesviridae* reactivation, 3 (10%) received corticosteroids (*p* = 0.53 for between groups comparison).

### Herpesviridae reactivation and outcomes

Outcomes of patients according to type of *Herpesviridae* reactivation (HSV alone, CMV alone, both viruses, or none) in each group (COVID-19 and influenza) are reported in Table 3: duration of MV, ICU length of stay and ICU mortality rate were similar in patients with or without *Herpesviridae* reactivation. ICU mortality rate of patients with HSV bronchopneumonitis was not different from ICU mortality of patients without HSV bronchopneumonitis (53% vs. 43%, respectively, *p* = 0.16 for between groups difference).

In univariable and multivariable analysis, *Herpesviridae* LRT reactivation (whatever the type of reactivation, i.e. HSV reactivation, CMV reactivation or both CMV and HSV reactivation) was not associated with ICU mortality in patients with COVID-19 and influenza (Additional file 1: Table S2).

## Discussion

In this study, we found that HSV and CMV reactivations in the LRT were frequent in patients with severe COVID-19, affecting 50% and 42% of patients, respectively. However, this high rate of *Herpesviridae* reactivation was close to that of patients with influenza-associated ARDS; taking into account death and extubation as competing events, HSV reactivation was more frequent in influenza patients than in COVID-19 patients, whereas rates of CMV reactivation were similar in both conditions. We also found that *Herpesviridae* reactivation (whatever the reactivation, i.e., HSV, CMV or both viruses) had no impact on outcomes.

To the best of our knowledge, only 6 studies have evaluated *Herpesviridae* reactivations in the blood and/or in the LRT of COVID-19 patients, and all found a high rate of *Herpesviridae* reactivation [5–10]. Results of these observational studies are summarized in Table 4. HSV was detected in the blood of 8–30% and in the LRT of 42–83% of ICU patients, whereas CMV was detected in the blood of 15–41% and in the LRT of 24% of ICU patients. Our results are in accordance with these reports and reinforce them, although the case mix was not similar (our study is the largest study on patients on MV, and most had ECMO support). In addition to HSV and CMV, some studies evaluated other *Herpesviridae*: Saade et al. found, that among 100 ICU patients (38 being immunocompromised), DNA of EBV was detectable in the blood of 58 [5]; Simonnet et al. found that among 34 ICU patients, EBV, and HHV-6 DNA were detected in the blood of their patients in 28 (82%) and 7 (22%), respectively [8].

**Table 4:** Studies having evaluated herpes simplex virus and/or cytomegalovirus reactivations in COVID-19 patients

Study	Population	Immunosuppresssion	HSV detection		CMV detection		Mortality
			Blood	LRT	Blood	LRT	
Franceschini [7]	70 patients 23 (33%) on IMV	3 (4.3%)	21 (30%)	–	29 (41%)	–	15 (21.4%)
Simmonet [8]	34 patients 30 (88%) on IMV	2 (6%)	–	–	5 (15%)	–	6 (18%)
Saade [5]	100 patients 54 (54%) on IMV	38 (38%)	12 (12%)	–	19 (19%)	–	28 (28%)
Seeße [6]	18 patients on IMV	3 (13.3%)	1 (7.7%)	15 (83%)	–	–	NR
Le Balc’h [10]	38 patients on IMV	3 (8%)	–	16 (42)	–	9 (24)	4 (10.5%)
Meyer [9]	153 patients 40 on IMV	NR	36/146 (24.7%)	19/61 (31.1%)	–	–	57 (37.3%)
Luyt, present study	145 patients on IMV 144 on ECMO	12 (8%)	5/19 (26%)	73 (50%)	15/29 (52%)	61 (42%)	63 (43%)

Results are expressed as n (%)

*COVID-19* coronavirus infectious disease 19, *HSV* herpes simplex virus, *CMV* cytomegalovirus, *IMV* invasive mechanical ventilation, *ICU* intensive care unit, *ECMO* extracorporeal membrane oxygenation, *LRT* lower respiratory tract, *NR* not reported

One of the originality of our work is to have compared patients with COVID-19 to patients with severe influenza. COVID-19 have been initially compared to influenza, since both virus may induce pneumonia and ARDS; however, it seems that COVID-19 is associated with significantly greater severity of illness, longer hospital stays, higher rate of ventilator-associated pneumonia and higher mortality rate [12, 24, 25]. As a matter of fact, in our patients, despite greater severity of influenza patients than COVID-19 patients (with higher severity scores at admission and higher rate of documented bacterial coinfection), the former spent less time on MV, on ECMO, and in the ICU. Whatever these differences, the rates of HSV and CMV reactivations in the LRT were close in the 2 groups, as well as the rate of HSV bronchopneumonitis. Although rates of HSV lung reactivation and HSV bronchopneumonitis were high in COVID-19 but also in influenza patients, they are similar to that of previous studies in different populations [18, 26]. Interestingly, despite 88% of our COVID-19 patients received corticosteroids vs. only 20% of influenza patients, the rate of Herpesviridae reactivations were close in both groups, suggesting that use of corticosteroids [mostly dexamethasone at a dosing of 6 mg/day during 10 days)] in COVID-19 patients may perhaps not be a risk factor for viral reactivation. However, our study was not designed to explore risk factors for Herpesviridae reactivation in COVID-19 patients; therefore, no formal conclusion on this particular point can be drawn.

In our study, 42% of patients with COVID-19 had CMV lung reactivation. Whereas CMV blood reactivation is well-described and occurs in roughly 30% of CMV-seropositive patients [3, 27], CMV lung reactivation has been poorly investigated. Moreover, the exact significance of CMV detection in the LRT remains controversial: apart in biopsies or autopsies findings, where CMV pneumonia has been diagnosed histologically [28, 29], no study has evaluated the relevance of CMV lung reactivation in ICU patients. In other words, whereas CMV lung detection is associated with lung disease is probable, but has never been confirmed.

Surprisingly, we found no impact on outcome of HSV or CMV reactivation, whereas previous studies have shown the opposite: indeed, HSV reactivation and bronchopneumonitis have been associated with increased mortality [18, 30], as well as CMV blood reactivation [3, 27, 30]. Moreover, although not reaching statistical significance, we observed a trend toward higher duration of MV and ICU length of stay in COVID-19 patients and *Herpesviridae* reactivation, as compared to COVID-19 patients without reactivation. This was previously described by others in similar patients, namely, VV-ECMO patients: Hraiech et al. found an association between *Herpesviridae* reactivation and duration of MV [31]. However, the population of the previous studies was not the same as ours, since we included patients with severe respiratory failure, almost all requiring ECMO support. In these patients, prognosis is probably driven more by the underlying condition (namely, ARDS and other organ failures) than by HSV or CMV reactivation. Similarly, in their randomized, double-blind, placebo-controlled study evaluating preemptive acyclovir, Luyt et al. found a positive effect of acyclovir on mortality only in the less severe patients, i.e., those with one organ failure or less at randomization, whereas acyclovir treatment had no impact on outcome in the most severe patients [32]. Last, the lack of power of our study may explain the difference between Hraiech study and ours. However, the significance of *Herpesviridae* reactivation and duration of MV is still matter of debate: it might be either a causative link (*Herpesviridae* reactivation has its own morbidity/mortality), or a bystander association (*Herpesviridae* reactivate in the most severe patients, with prolonged duration of MV).

Our study has several limitations that should be underlined. First is its retrospective monocenter design that included the most severe COVID-19 patients, almost all of whom requiring VV-ECMO, making our results difficult to extrapolate to other ICUs with different case-mixes. However, the frequencies of HSV and CMV detection found here were close to those previously reported. Second, we included only patients on MV, because HSV and CMV lung sampling are performed only when a patient is suspected of having developed VAP. Therefore, we are not sure that patients who were not sampled did not have HSV or CMV lung reactivation, and our results cannot be extrapolated to patients without MV. Third, we included patients who had HSV and CMV testing in BAL: 44/134 (33%) influenza patients and 119/264 (45%) COVID-19 patients were not tested for *Herpesviridae*, which is a selection bias. Therefore, the frequencies of HSV or CMV lung reactivations given in the present study may not be representative of those occurring in all ICU patients. Fourth, HSV and CMV serologic status of our patients is not known. Since it is obvious that rate of HSV or CMV reactivation depends on the rate of positive serologic status, our results are, therefore, not applicable in a different population with different serologic status. Fifth, COVID-19 and influenza patients were not strictly comparable, since the latter were more severely ill at admission, had more bacterial coinfection and spent less days on MV and ECMO; therefore, the comparisons or *Herpesviridae* reactivations between the 2 groups may be inappropriate. Sixth, we acknowledge that our study lacks of power, as well as lack of correction of alpha risk for multiple comparisons. Therefore, difference between the 2 groups may occur and not be detected. Finally, only a small proportion of our patients had HSV or CMV blood testing. Therefore, the exact frequencies of these blood reactivations in COVID-19 or influenza patients cannot be determined, as well as their impact on outcome.

## Conclusions

HSV and CMV lung reactivation are frequent in COVID-19 patients, but as frequent as in patients with influenza-associated severe pneumonia. HSV bronchopneumonitis is also frequent in COVID-19 patients, but similar to that of previously described frequency. HSV or CMV lung reactivations are not associated with impaired outcome, as compared to patient without HSV or CMV reactivation, but probably because our study included the most severe patients. Whereas an antiviral treatment may improve outcome in these patients remains to be determined.

## Supplementary Information

**Additional file 1: Table S1. **HSV and CMV blood reactivation. **Table S2.** Univariable and multivariable analysis of factors associated with intensive care unit mortality.

## Data Availability

The data sets generated during the current study are available from the corresponding author on reasonable request.

## Abbreviations

ARDS: Acute respiratory distress syndrome
ARF: Acute respiratory failure
BAL: Bronchoalveolar lavage
COVID-19: Coronavirus infection disease 2019
ECMO: Extracorporeal membrane oxygenation
ICU: Intensive care unit
MV: Mechanical ventilation
SAPS: Simplified Acute Physiology Score
SARS-CoV-2: Severe Acute Respiratory Syndrome coronavirus 2
SOFA: Sequential organ failure assessment
VAP: Ventilator-associated pneumonia
WBC: White blood cell
