# Supplementary material for: Herpesviridae lung reactivation and infection in patients with severe COVID-19 or influenza virus pneumonia: a comparative study
Source: Ann Intensive Care. 2022 Sep 24;12:87. doi: 10.1186/s13613-022-01062-0 (PMC9509504; doi:10.1186/s13613-022-01062-0)
Supplement: Supplementary file 1 — Additional file 1: Table S1. HSV and CMV blood reactivation. Table S2. Univariable and multivariable analysis of factors associated with intensive care unit mortality. [file 13613_2022_1062_MOESM1_ESM.docx]

**Herpesviridae lung reactivation and infection in patients with severe Covid-19 or influenza virus pneumonia: a comparative study**

CE Luyt, S Burrel, D Mokrani, M Pineton de Chambrun, D Luyt, J Chommeloux, V Guiraud, N Bréchot, M Schmidt, G Hekimian, A Combes, D Boutolleau

Additional file

**Results**

HSV DNA was tested in blood of 31 patients (19 with Covid-19 and 12 with influenza), and was recovered only in 5 patients with Covid-19 (all 5 had HSV lung reactivation) (Table S1).

CMV was tested in blood of 64 patients (29 Covid-19 patients and 35 influenza patients), and was recorded in 15/29 (52%) and 12/35 (34%) patients, respectively (p = 0.8 for between groups comparison) (Table S1).

Table S1. HSV and CMV blood reactivation

| Characteristic | Covid-19 Patients  n = 145 | Influenza Patients  n = 89 |
| --- | --- | --- |
| HSV blood reactivation | 5/19 (26) | 0/12 |
| HSV blood load, copies/ml | 1,184 (863–266,459) | – |
| Time from MV start to HSV blood reactivation, days | 17 (14–20) | – |
| CMV blood reactivation | 15/29 (52) | 12/35 (34) |
| CMV blood load, IU/ml | 262 (125–5,961) | 449 (144–1,519) |
| Time from MV start to CMV blood reactivation, days ^c^ | 26 (16–57) | 16 (8–24) |

Results are expressed as n (%) or median (IQR). Abbreviations: Covid-19, coronavirus infectious disease 19. HSV, herpes simplex virus. BAL, bronchoalveolar lavage. CMV, cytomegalovirus. MV, mechanical ventilation. IU, international unit

Table S2. Univariable and multivariable analysis of factors associated with intensive care unit mortality

|  | Univariable analysis  OR (95% CI) | Multivariable analysis ^a^  OR (95% CI) |
| --- | --- | --- |
| Overall population (n =234) | | |
| Age, per year | 1.06 (1.04–1.09) | 1.07 (0.04–1.1) |
| Immunosuppressed | 2.81 (1.16–6.8) | 2.59 (0.94–7.13) |
| SOFA score at ICU admission, per point | 1.17 (1.08–1.25) | 1.18 (1.09–1.27) |
| Herpesviridae reactivation |  |  |
| No herpesviridae reactivation  HSV reactivation  CMV reactivation  Both HSV and CMV reactivation | 1  1.06 (0.55–2.03)  1.17 (0.47–2.92)  1.57 (0.81–3.06) | 1  0.69 (0.32–1.45)  1.39 (0.49–3.23)  1.52 (0.71–3.23) |
| Covid-19 patients (n = 175) | | |
| Age, per year | 1.09 (1.05–1.14) | 1.11 (1.06–1.17) |
| Immunosuppressed | 4.39 (1.14–16.96) | 8.46 (1.62–44.06) |
| SOFA score at ICU admission, per point | 1.19 (1.07–1.33) | 1.18 (1.06–1.32) |
| Herpesviridae reactivation |  |  |
| No herpesviridae reactivation  HSV reactivation  CMV reactivation  Both HSV and CMV reactivation | 1  0.89 (0.36–2.19)  0.98 (0.34–2.82)  1.71 (0.75–3.91) | 1  0.65 (0.22–1.88)  0.84 (0.25–2.85)  1.08 (0.4–2.93) |
| Influenza patients (n =89) | | |
| Age, per year | 1.04 (1.01–1.08) | 1.05 (1.01–1.1) |
| Immunosuppressed | 1.78 (0.53–5.93) | 1.25 (0.33–4.74) |
| SOFA score at ICU admission, per point | 1.15 (1.03–1.29) | 1.23 (1.08–1.4) |
| Herpesviridae reactivation |  |  |
| No herpesviridae reactivation  HSV reactivation  CMV reactivation  Both HSV and CMV reactivation | 1  1.16 (0.43–3.12)  3.69 (0.34–39.85)  1.35 (0.44–4.18) | 1  0.76 (0.25–2.32)  8.49 (0.54–134.52)  2.39 (0.62–8.82) |

Abbreviations: OR, odds ratio. HSV, herpes simplex virus. CMV, cytomegalovirus. Covid-19, coronavirus infectious disease-19.
